# A Comprehensive Review on Bio-Nanomaterials for Medical Implants and Feasibility Studies on Fabrication of Such Implants by Additive Manufacturing Technique

**DOI:** 10.3390/ma13010092

**Published:** 2019-12-23

**Authors:** Rajkumar Velu, Theo Calais, Arunkumar Jayakumar, Felix Raspall

**Affiliations:** 1 Digital Manufacturing and Design Centre (DManD), Singapore University of Technology and Design, Singapore 486842, Singapore; theo_calais@sutd.edu.sg (T.C.); felix.raspall@uai.cl (F.R.); 2 Sustainable Solutionz, Chennai 600017, India; arunkumar.h2@gmail.com

**Keywords:** additive manufacturing, 3D printing, nanocomposites, medical implants, 3D printed bone, 3D printed denture, prosthetics

## Abstract

Nanomaterials have allowed significant breakthroughs in bio-engineering and medical fields. In the present paper a holistic assessment on diverse biocompatible nanocomposites are studied. Their compatibility with advanced fabrication methods such as additive manufacturing for the design of functional medical implants is also critically reviewed. The significance of nanocomposites and processing techniques is also envisaged comprehensively in regard with the needs and futures of implantable medical device industries.

## 1. Introduction 

The unique attributes of nanomaterials are their higher surface area, reactivity, and robustness compared to their bulk counterparts. Their integration into matrixes has demonstrated the possibility to enhance the mechanical, chemical, and physical properties of the resulting composites. As a consequence, they have been intensively studied for a wide range of engineering applications, such as energetic materials [1], biomedical applications [2], microelectronics [3], etc. Among these fields, the design of innovative and multifunctional implants and tissue engineering are of primary interest due to their life-saving nature. Implants, such as artificial pacemakers and cochlear implants, are used to regulate the function of human mechanisms when abnormalities occur [4]. These materials must combine high strength-to-weight ratio, high surface area, and biocompatibility. In addition to the identification of a suitable combination of material properties, it is also critical to achieve a favorable topography for cell attachment, proliferation, and differentiation, together with biocompatible surface chemistry [5]. In this regard, the incorporation of nanomaterials into matrixes (polymers, ceramics, or metals) faces several challenges, such as a controlled and homogenous volume fraction of the filler inside the matrix.

Additive manufacturing (AM) [6], or 3D-printing, is an emerging technique that is extremely promising for biomedical applications, due to its potential to achieve complex and compact structures adaptable to the anatomy of patients [7,8]. Beyond these aspects, the geometric freedom, low volume production, and environmental sustainability of AM [9,10] represent significant advantages for biomedical industries. However, the processable materials remain limited to thermoplastics (e.g., ABS, PLA, etc.) and metal alloys (e.g., Ti-64, Ti-Ni, etc.). In the last decade, materials scientists have been developing innovative composite materials adapted to the layer-by-layer building of objects for a large range of applications, such as aerospace, automotive, and biomedical applications [11,12]. A significant effort has been made to enhance their functionality via the incorporation of fillers and nanofillers [13]. However, only a few reviews have focused on nanocomposite fabrication based on additive manufacturing for biomedical applications [7,13]. Here, a comprehensive study of bio-nanocomposites manufactured by AM technologies is presented, with a focus on medical implants. The review is divided into six main sections. The composition of current nanocomposites used for medical applications and standard mixing processes are presented in Section 2 and Section 3, respectively. Additive manufacturing technologies and their impact on current technologies are reviewed in Section 4. The effects of fillers on mechanical and physical properties of nanocomposites and the use of AM for the design of advanced biocompatible medical implants are discussed in Section 5. Finally, current limitations of additive manufacturing for manufacturing medical implants are identified in Section 6, before a conclusion on medical industry needs and future directions for the design of implantable devices in Section 7. 

## 2. Nanocomposites Used for Biomedical Applications

### 2.1. Materials Compatible with Medical Implants

To be used as implants, materials (metals, ceramics, or polymers and their composites) have to be prudently selected and engineered to combine biocompatibility with specific properties depending on the end-use of the device, such as density, elasticity, fracture and wear resistance, etc. Historically, metals have been predominantly used as implants due to their exceptional strength and ductility [14]. In particular, stainless steel, CoCr alloys, and Ti alloys are widely used because of their high corrosion resistance. Further surface functionalization is often required to enhance the biocompatibility with organic tissues [15], e.g., by coating implants with hydroxyapatite (Hap) for bone compatibility [16]. Noble metals, such as Au, Ag, and Pt are interesting candidates for the dentistry application due to their castability and ductility. 

Polymers with biodegradable and biocompatible properties, such as chitosan, gelatin, heparin, and collagen, have been extensively used for tissue engineering and regenerative medicine [17]. However, these materials are weak to the human body’s immune response and degrade over time. Regarding the synthetic polymers, aliphatic polyesters, such as polylactide, poly-ε-caprolactone (PCL), polyglycolide and their copolymers, are widely used as non-permanent scaffolds due to their non-toxicity, biocompatibility, biodegradability, and substantial mechanical properties [18]. Poly (ethylene glycol) (PEG) is also a renowned biocompatible polymer with hydrophilicity and solubility over a wide range of solvents, facilitating its processing. Poly (vinyl alcohol) (PVA) and poly (acrylic acid) (PAA) have also been used, but their non-degradability limits their usage in implants. Polyurethanes have also been studied due to their high mechanical strength. It is possible to increase their biodegradability by adding hydrolysable polymeric segments such as PCL [19]. In addition, the biocompatibility of some conductive polymers, such as polypyrrole, polyaniline, polythiophene, and their derivatives, has been well established, thus extending their applicability to electrically stimulated tissues, such as nerve, bone, muscle, cardiac cells, etc. [20]. However, these materials have poor mechanical properties, are hydrophobic, and are non-degradable, thus requiring the design of novel composites with multifunctional attributes.

Bio-ceramics are of vital importance for medical implants and for tissue engineering applications [21]. For example, TiO_2_ has attracted a lot of attention as a scaffold for bone regeneration due to its biocompatibility, capability to enhance the ingrowth of bone and vascular tissues, extensive antimicrobial activities, and osteoconductivity [22]. Calcium phosphates are also an ideal choice as they can replicate the configuration of bones [23]. 

These bulk materials have their own pros and cons, depending on the targeted application. Combining these materials with nanomaterials can enhance or tune their mechanical performances or their functionality. Different types of nanomaterials can be used for biomedical applications, depending on the environmental conditions of the targeted device. In essence, we can define nanocomposites as a matrix (most often polymers), with specific structural properties, doped with fillers with dimensions lower than 100 nm. The role of fillers is to modify the structural properties of the matrix or to add functionality, such as electrical conductivity, biocompatibility, etc. The use of nanoscale fillers is particularly interesting to increase the magnitude of the change in the properties of composites. However, the challenge lies in their appropriate dispersion into matrixes to assure the maximization of the properties and to limit their aggregation. 

### 2.2. Nanocomposites with Metallic-Based Nanofillers

The use of metallic nanofillers (e.g., gold or silver nanoparticles) is interesting for biomedical applications to enhance the electrical conductivity or antimicrobial properties of tissues. For example, the incorporation of gold nanoparticles into extracellular matrixes (ECM) demonstrated the enhancement of the cellularity by reinforcing the ECM, while mitigating the inflammation for musculoskeletal tissue engineering applications [24]. Silver, in the form of nanoparticles, is also well recognized for its antimicrobial properties, specifically as an alternative to antibiotic drugs. Bhowmick and Koul designed a PVA-based hydrogel doped with Ag nanoparticles that was able to sustain a microbial environment for 96 h [25]. This hydrogel appeared to be an interesting candidate to dress wounds and restrain microbial activity. Similarly, bio-based polyurethane backbones have been functionalized with magnetic particles (Fe_2_O_3_), resulting in a material with both antibacterial and magnetic properties [26]. Kumar et al. reported the use of graphene oxide as an intermediate to reinforce PCL doped with silver [27]. PCL composites with both graphene oxide and silver showed an increase in the modulus, electrical conductivity, sustainable release of Ag ions, and non-toxicity with human cells in comparison with PCL doped with Ag nanoparticles alone.

### 2.3. Nanocomposites with Ceramic Nanofillers

A typical example of the reinforcement of polymers with ceramic nanofillers could be the reinforcement of chitosan with Hap nanopowders [28,29,30]. Pure chitosan has poor mechanical properties, thus excluding its use in load-bearing applications. The use of Hap with a crystal structure close to that of natural bone allowed the synthesis of scaffolds with controlled pore structures with high strength [29]. Similar to chitosan, cellulose has been functionalized with nano-Hap to produce artificial bone tissue scaffolds [31,32]. Some researchers have studied the impact of fillers such as zinc on Hap to enhance the cytocompatibility and the corrosion resistance of metal implants [33]. TiO_2_ nanoparticles are also widely used as fillers for the reinforcement of polymers. For example, Kiran et al. reported the functionalization of PCL with TiO_2_ nanoparticles (which possess an antibacterial properties) to bioactivate titanium implants by coating, and thus favoring the cell attachments [34]. Similarly, Khandan et al. used diopside to increase the bioactivity, wettability, and hardness of bovine-hydroxyapatite incorporated as a coating for titanium implants [35]. The reinforcement of rubber with fabrics is also critical to manufacture of surgeon gloves, warm water bags, and many other devices with high mechanical performance and strong resistance to bacteria. For example, Li et al. combined ZnO particles and cellulosic fibers to mechanically reinforce rubber composites along with antibacterial properties [36]. In addition to the antibacterial properties, ZnO is used as a dispersing agent to avoid the agglomeration of cellulosic fibers into the rubber matrix.

### 2.4. Nanocomposites with Carbon-Based Nanofillers

Carbon nanotubes (CNTs) have been extensively used for doping polymers, such as polyurethanes [37], PCLs [38], or biopolymers such as collagen [39] or chitosan [30,40]. In addition, to the mechanical reinforcement, the incorporation of CNTs on biocompatible polymers increases the growth and differentiation of different cells such as bone, neurons, etc. [41], with promising results for osteogenesis. For example, the integration of single-walled carbon nanotubes (SWNTs) into chitosan doped with hydroxyapatite improved the osteoblast adhesion to scaffolds [40]. However, the cytotoxicity of CNTs has to be fully addressed before implementation in the body [42].

Graphene is another carbon-based material intensively studied for biomedical applications. It can be found in three configurations: (i) unoxidized pure graphene sheets, (ii) graphene oxide (GO), and (iii) reduced graphene oxide (rGO). For example, Kumar et al. used GO to functionalize the biopolymer polypyrrole to serve as a coating for implants with improved biocompatible characteristics [43].

### 2.5. Nanocomposites with Cellulose-Based Nanofillers

Cellulose-based nanomaterials are gaining increased interest due to their large availability, mechanical properties, ability to self-assemble in network structures, biocompatibility, and low cytotoxicity [44,45,46,47], making them potential candidates for biomedical applications [48]. However, their hydrophilic nature challenges their dispersion in polymer-based matrixes [44]. Cellulose fillers can be classified into three categories: (i) cellulose nanocrystals (CNs), (ii) cellulose nanofibrils (CNFs), and (3) bacterial cellulose (BC) [48]. The use of CNs as additives in cement-based materials have shown potential for improving mechanical strength [49,50,51]. The biocompatibility and biodegradability of CNs are also interesting for biomedical applications, such as tissue repair and healing (skin, bones) and medical implants. Bacterial-based cellulose materials are frequently used for vascular implants [52,53]. Bacterial cellulose is composed of a pure cellulose nanofiber mesh spun by bacteria and presents remarkable strength with the ability to be engineered at the nano, micro, and macroscales [54]. In particular, the biocompatibility, optimal three-dimensional and microfibrillar structure, and physical barrier to reduce bacterial infection are some of the numerous advantages of BCs for the synthesis of nanocomposites for biomedical applications [53]. Their usefulness has been reported for numerous applications, such as bone and cartilage regeneration (e.g., BC/Hap nanocomposites [55,56,57]), dental grafting, artificial cornea (e.g., BC/PVA composites [58]), wound dressing (e.g., BC/hyaluronan composite films [59]), etc. Polyurethane-based nanocomposites doped with nanocellulose have been synthesized for prosthetic vascular grafts [45], exhibiting high elongation at break (800–1200%) and an ability to withstand hydraulic pressures up to 400 kPa. Polyvinyl alcohol (PVA), another hydrophilic biocompatible polymer, has also been extensively used for soft tissue replacement, such as heart valve tissue [60].

## 3. Traditional Methods for Nanocomposite Synthesis for Biomedical Applications

### 3.1. Sol-Gel Technologies

The most widespread technique for the preparation of nanocomposites is the sol-gel technique, i.e., the transition from a precursor solution (sol) containing monomers and subsequent gelification (gel) of the chemical species into a solid form. This strategy has the advantage to facilitate the homogenous dispersion of particles into the polymer, either by dispersion into colloidal suspension before gelification or by growth of particles in situ during the gel step by adding the suitable precursors to the nucleation of fillers. The polymer formation based on the solvent evaporation facilitates the shaping of the final composite into thin films.

For example, cellulose nanoparticles are usually obtained in stable aqueous colloidal suspensions and can be easily dispersed in hydrosoluble polymers. Then, the evaporation of the solvent leads to the solidification of the matrix with a homogenous dispersion of cellulose nanoparticles. Casting, freeze-drying, and hot pressing are conventional methods for shaping these cellulose-based nanocomposites. A large amount of polymer-based composites reinforced with cellulose fillers have been reported [45]. For example, Butron et al. dissolved poly(ethylene brassylate) (PEB), a polymer similar to polycaprolactone, in chloroform, before mixing cellulose nanocrystals into the suspension by sonication [61]. Composite films were then manufactured by casting and hot-pressing (175 °C, 250 bar) the dispersion in moulds. Similarly, Das et al. dispersed Fe_2_O_3_ nanoparticles in chloroform and then mixed the colloidal suspension to the bio-based polyurethane before the second step of the polymerization of the polyurethane [26]. Rashti et al. reported the use of sol-gel method for the synthesis of biocompatible PU doped with silica nanoparticles, thus improving mechanical and biocompatibility characteristics [62]. In addition, conjugation, or cross-linking is generally used to assure covalent grafting between nanoparticles and tissues and thus improving the integration of nanofillers within matrixes. For example, Smith et al. conjugated gold nanoparticles with a porcine extracellular matrix by first grafting 2-mercaptoethy-lamine (MEA) on gold nanoparticles before conjugation with 1-ethyl-3-[3-dimethylainopropyl] carbodiimide (EDC) [24]. 

Another strategy is the growing of nanomaterials in situ. Ribeiro et al. synthesized a silk fibroin/nanoHAp hydrogel by synthesizing in situ silver and gold nanoparticles from metallic salts. These tissues exhibited significant inhibition ability against bacterial activity without compromising the cell behavior, making them interesting candidates for bone tissue engineering [63]. In this case, the reduction of silver and gold ions in solution takes place directly on the substrate. In another study, Kim et al. synthesized Hap doped with TiO_2_ particles by dissolving calcium- and phosphate-based salts in ethanol solution in one hand, and by dissolving titanium prop oxide with ethanol solution on the other hand [64]. The mixtures of the two solutions in different ratios resulted in HA-TiO_2_ nanocomposites with enhanced strength (~50% enhancement) and bioactivity. Cai et al. prepared a composite based on a chitosan-PLA matrix doped with HA particles by in situ syntheses [65].

### 3.2. Thermally Induced Phase Separation (Freeze-Drying)

Freeze-drying methods have gained considerable attention for the synthesis of foams or porous structures for a wide range of materials, due to the ease of process and the possibility to tune the pore size and direction [66]. Several teams reported the use of this technique for the synthesis of bio composites for tissue engineering, such as nano-HA/collagen/PLLA composites [67,68,69].

### 3.3. Electrospinning

Electrospinning is a simple technique for porous nanofiber fabrication. A wide range of materials can be spun, resulting in a hierarchical assembly within the sub-micron range and with functional properties [70]. The subsequent fibers have a porous structure suitable for cell development, offering support with satisfying rigidity, and are thus appropriate for biomedical and tissue engineering applications [71,72]. For example, Asran et al. synthesized PVA nanofibers and nanocomposites doped with Hap by electrospinning techniques, resulting in an increase in the rigidity of the scaffolds [73]. Electrospinning has been widely used to replicate the anisotropic nature of cardiac cells. For example, Puperi et al. used the combination of electrospun polyurethane with a PEG-based hydrogel to recreate the heterogeneous structure of a heart-valve tissue [74]. Xue et al. studied the impact of the polymer formulation on the fiber morphology and the biaxial mechanical properties of elastomeric fibrous scaffolds made of PEG-based hydrogels and PCL blends [75]. Ravichandran et al. analyzed the potential of a core-shell nanofibrous cardiac patch as a regenerative technique after myocardial infarction [76].

## 4. Rapid Prototyping and Additive Manufacturing Methods 

Additive manufacturing, also commonly called 3D printing, is an advanced fabrication method building three-dimensional components (usually designed using computer-aided design (CAD) models) by deposition of materials layer by layer [77]. The unique benefit of this technology is the possibility to fabricate highly complex structures with constrained geometries, which cannot be fabricated by the subtractive process [78]. This technology is widely used for rapid prototype modelling [79]. It facilitates the quick and efficient assessment of a concept through rapid mock-up fabrication. The rapid prototyping process may be repeated many times until the component meets a variety of demands, including cost-effectiveness, compliance requirements, and user needs. As a consequence, numerous cutting edge research studies have focused on the fabrication of end-use products based on AM technology [80,81]. 

Different types of AM systems can be distinguished as a function of the nature of the material being processed. For example, powder fusion, extrusion, and liquid polymerization are based on the use of powder, solid-liquid-solid transitions, or liquid-solid transitions, respectively [82]. In each category, different technologies can be used. For example, selective laser sintering (SLS), selective laser melting (SLM), and electron beam melting (EBM) are based on the fusion of powder. Fused deposition modelling (FDM) is based on the material extrusion (fusion then solidification of the material). Finally, stereolithography is based on the polymerization of a liquid (liquid-solid transition). All these techniques have their own operational materials, processing system, and layer creation technique [83]. Novel materials with unique combinations of properties can be designed using these techniques [84,85]. 

**Figure 1:**
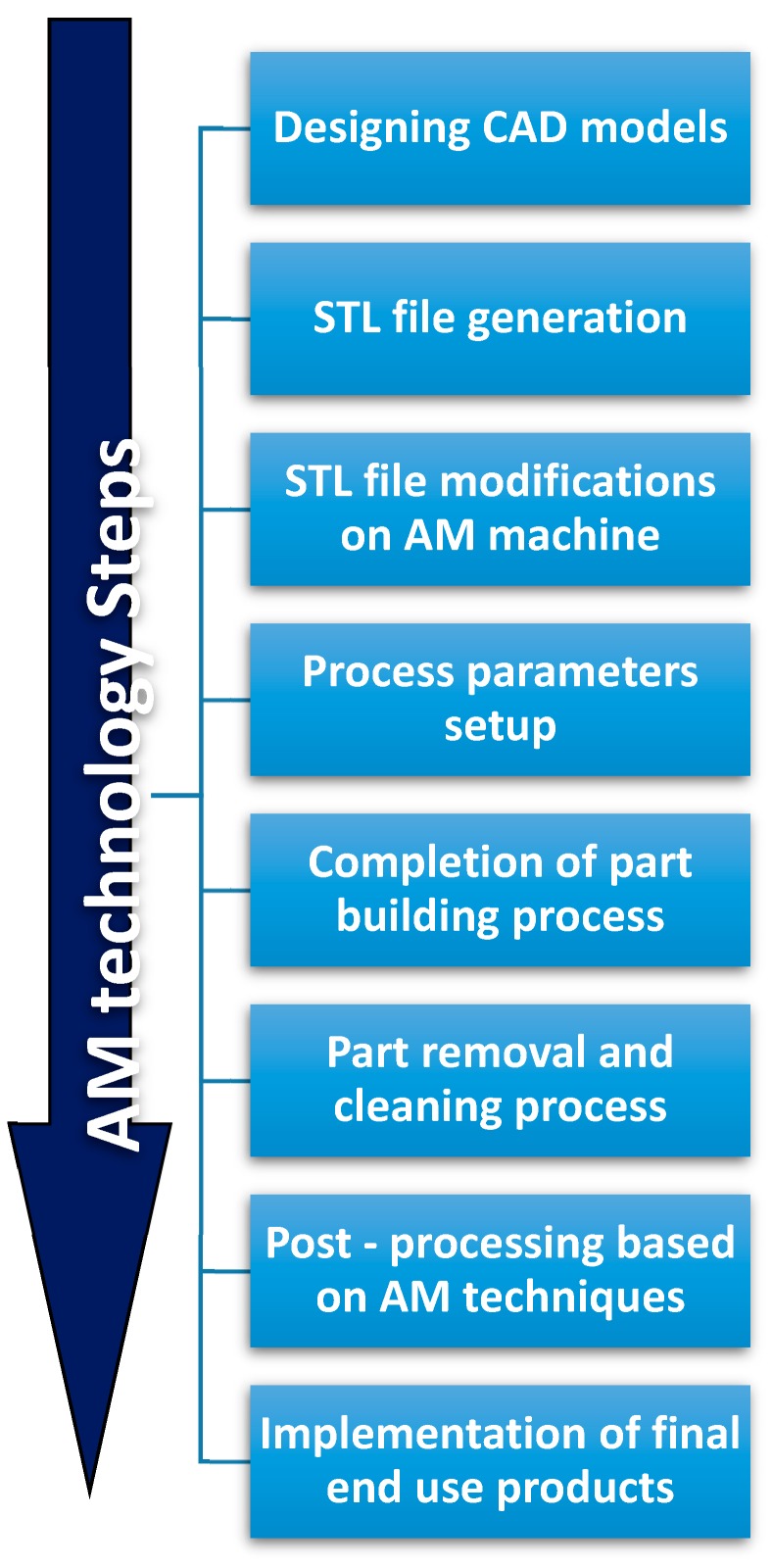
Additive Manufacturing processing steps.

The development of AM technologies could significantly impact other non-technical areas. For example, health and life quality could be improved by the possibility to customize health products to everyone. The environmental impact of manufacturing technologies could be reduced by developing sustainability in the design/production of consumables, or by simplifying supply chain systems. Monitoring and measuring AM processes using Internet of Things (IoT), sensors, and models, could help to reach these objectives [86,87,88]. Currently, the long sequence of AM procedures illustrated in Figure 1, from the conceptual design to the final end-use product [77], limits large-scale application of AM. Development and commercialization of novel materials with uncommon properties and functionalities are also required for 3D-printing systems. In particular, the biomedical field could greatly benefit from these developments: medical models are characterized by individualized anatomical structures, complex geometries, and multifunctionality. Therefore, they require a systematic design methodology particularly suited with AM technologies [89]. 

**Figure 2:**
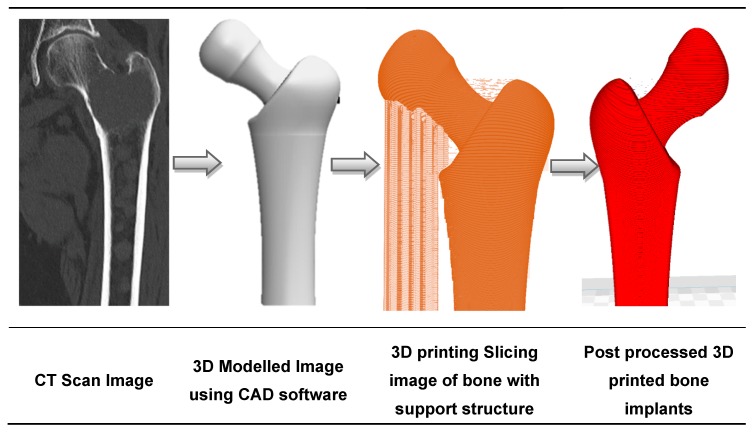
The sequence of bone implant fabrication using additive manufacturing.

Disrupted by the emergence of AM along with traditional computerized tomography (CT) scanning techniques and CAD modelling, the medical implant industry has significantly evolved in the past decades. Initially used for visualization and diagnosis purposes, 3D imaging data has been combined with AM to build medical models adapted to the patient’s morphology [90,91]. This is illustrated in Figure 2, with the design of a hip implant using AM [92]. Medical experts visualize the patient’s injuries in 3D, and are thus able to simulate preoperative surgical procedures and model and manufacture artificial implants [93]. Beyond the design of implants, reconstructed 3D-models have been used to study the pathophysiology of a disease. For example, Pasta et al. used a 3D printed mock-up of the aorta to study the hemodynamics of a vessel through a circulatory flow loop [94,95]. 

Gibson et al. described the materials processed by AM technologies for medical applications [96]. Only a few polymers were identified as safe for being placed inside the human body. Regarding metals and alloys, titanium is usually used to fabricate implants due to its biocompatibility [97,98]. However, such hard-metallic materials require specific technologies to be processed, justifying the need for a less expensive manufacturing process. Zinc is another biocompatible metal suitable for the manufacture of bioabsorbable cardiac stents due to its corrosion behavior [99].

The evolution of materials used in AM technologies could open possibilities in numerous domains for biomedical implants, such as customized prosthetics, tailor-made implants, functional implantable devices, drug delivery, and tissue engineering [100,101]. For example, the orthopedic implant industry has been revolutionized by the use of AM technology, notably for the production of standard-sized implants [102]. The most widely used AM processes for orthopedic implants are SLM and EBM technologies [103,104]. The SLM process allows the easy fabrication of complex mesh structures [105], leading to innovative implants with reduced production cost and lead times compared to those of traditional fabrication processes [106]. Despite these fabrication advantages, the complexity of the implant is inherently linked to the ability to mimic bone structure, with the porosity and textured surface inducing high friction and bone ingrowth around the implant [107]. 

The total additive manufactured implants fabricated from 2014 to 2026 is projected to increase drastically, as reported by Smartech publishing in 2017 [108]. The material selection for manufacturing a specific biomedical product for a patient is not an easy task, particularly for advanced materials with significant and controlled properties. Generally, commonly used materials, such as high-performance polymers, metals, ceramics, or even biomaterials, have the required strength, rigidity, and heat resistance, but substantial modifications could improve their properties or increase their functionalities. Thus, polymers and composites are the most suitable and employable materials for AM-fabricated biomedical devices [109]. 

## 5. Additive Processing of Nanocomposites for Medical Implants 

The breadth and depth of implant research and development of AM for nanocomposite materials have led to the inevitable crossover of the two emerging fields [110]. The interest and investment in this interdisciplinary field can be justified by the raised awareness of the biological impacts of traditional manufacturing processes. Indeed, it seems logical to combine the material optimization of AM with biocompatibile composites for biomedical implants production. Additionally, bioinspired nanocomposites have been designed either to exhibit advanced functionalities, such as adhesive films, super hydrophobicity, and photonic coatings, or to mimic a specific biological function [111,112]. The development of nanocomposites for the fabrication of prosthetic devices, implants, drug delivery, and tissue engineering have achieved a significant impact on both biomedical fields and AM technology [6,113,114]. Many nanomaterials are biocompatible and biodegradable, which makes them particularly appealing for bioprinting applications and the subsequent enhancement of the desired properties of the final end-use product [115,116]. Other integral properties, such as physical, chemical, mechanical and optical properties are also influenced by the use of nanoscale fillers [117]. 

**Figure 3:**
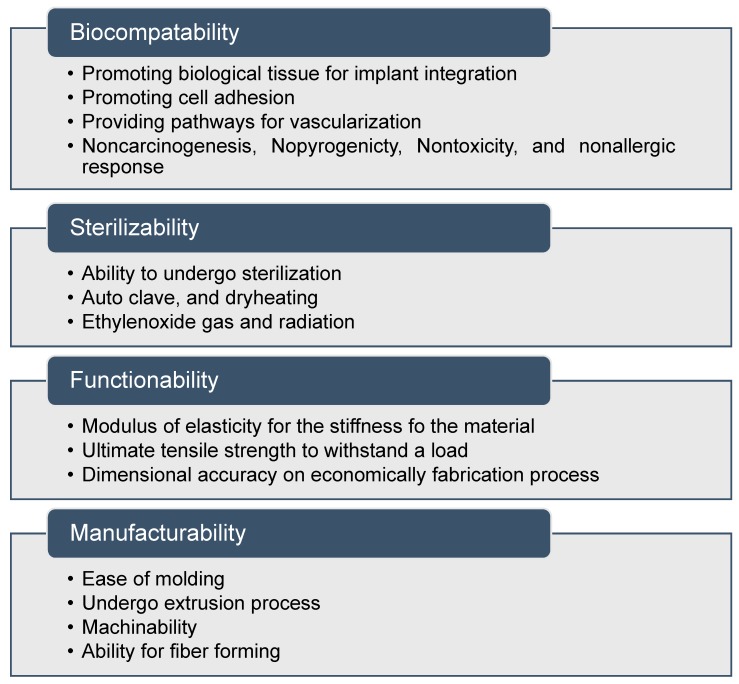
Properties of nanocomposite materials for medical applications.

In the context of biomedical implant fabrication, the term bio-nanocomposites is adopted, corresponding to the mixing of organic fillers with polymer matrices [118]. In general, matrices include natural polymers such as collagen, gelatin, enzymes, polypeptides, and polynucleic acids. Nanofillers act as molecular bridges within the matrix to attain the required properties [119,120,121]. Osteogenic differentiation has been stimulated using nanoscale surface topographies with feature sizes below 100 nm. The nanocomposites exhibit several biocompatibility, sterilizability, functionality, and manufacturability features, and can be classified as a medically graded material, as shown in Figure 3 [122,123]. Optimizing the suitable shape, size, and aspect ratio of the nanoscale features is the main challenge to improve 3D printed biomaterials.

Table 1 provides a systematic summary of the top 3D printed implantable devices based on nanocomposites: orthopedic implants, prosthetic devices, spinal rods, and bone plates. These orthopedic and prosthetic device fabrication methods have been rapidly adopted by users in the medical industry. Prostheses replace missing body parts lost through trauma, disease, or congenital conditions. In the US alone, an estimated 1.7 million people rely on prosthetics; this figure is projected to double by 2050 as a result of longevity and the prevalence of diabetes.

Nanocomposites manufacturing using AM technologies for implants needs specific attention from the scientific community. In order to ensure the safety of the newly developed biomaterials, it has become a prime focus to validate their physical properties, chemical stability, and biocompatibility along with their toxicological profile [137]. Tests on human subjects following legal and ethical considerations are mandatory to evaluate the biocompatibility of these novel materials. Therefore, to provide therapeutic solutions for various human diseases and permanent replacement of diseased tissues, clinical tests, such as cytotoxicity, histotoxicity, or genotoxicity, must be conducted, as recommended by the ISO (International Organization for standardization) [138,139]. ISO and FDA (Food and Drug Administration) have published the appropriate protocols, guidelines, and standards for the biocompatibility evaluation of newly developed materials [140]. 

**Figure 4:**
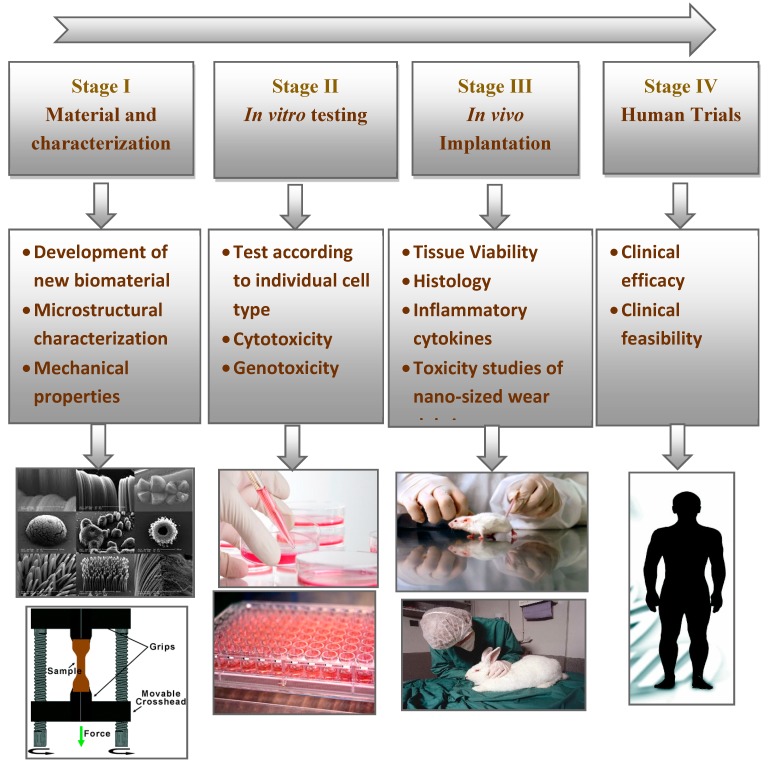
Steps involved in the translation of newly developed biomaterials.

Consequently, to convert a biomaterial into osseous systems, ISO standards recommend the methodology shown in Figure 4. The initial stage is to identify the material by considering positive and negative controls, extraction conditions, and choice of cell lines and cell media. The characterization of the material chemistry should also be analyzed. The next stage is the evaluation of in vitro and in vivo performances to avoid anomalies or potential toxicities. These studies should also follow the good laboratory practice (GLP) regulations. Once the series of in vitro tests are screened and approved, the newly developed material should undergo clinically relevant in vivo osseointegration tests. In particular, the in vivo tests involve implantation into an animal model to evaluate its histocompatibility. Then, the material is approved from the institutional committee for clinical trials in human patients. 

First, the in vitro tests assess the cytotoxicity of the selected nanocomposite. The cytotoxicity of the material to a specified cell type can be studied either by directly seeding the cells on the surface of the material or by exposing the cells to the extraction fluid. These tests are also known as indirect toxicity evaluations [141]. However, selecting the appropriate assay always impacts the evaluation of the cytotoxicity of the materials [142]. In addition, the other crucial parameters to be considered are cell lines, controls, biochemical assay types and culturing time. There are different types of cell lines for each system. For nerve regeneration, Schwann cells and neuroblastoma cells are used to evaluate the in vitro cytotoxicity of the materials [143]. Similarly, for orthopedic implant materials, human fetal osteoblast or osteosarcoma cell lines are used to check the cellular compatibility [144,145]. Keratinocytes or fibroblasts are used for determining the cytotoxic potential of wound dressing materials [146].

Xia et al. investigated macrostructure, morphology and mechanical strength of biomimetic composite scaffold using a SLS process with nano-HAp/PCL [147]. First, it is common to observe porous structures when using SLS processes. Indeed, gas bubbles can be formed in the melt pool of the polymer matrix. The authors also observed porous scaffolds with a porosity range from 70% to 78%. The results revealed that the level of attachment and proliferation of cells on porous nano-HA/PCL was significantly increased compared to neat PCL scaffolds. The scaffolds were further analyzed by in vivo studies with implantation into rabbit femur defects for 3, 6, and 9 weeks. Results revealed that both nanocomposites and pure PCL had good biocompatibility, but the nanocomposites enhanced the formation of new bone.

Similarly, nanosized osteoconductive calcium phosphates including HA, tricalcium phosphate (TCP), and substituted HA have attracted much attention in biomaterials due to their smaller size, high surface-area-to-volume ratio, and similarities with natural bone when combined with natural and synthetic polymers. Bin Duan et al. fabricated 3D nanocomposite scaffolds based on calcium phosphate (Ca-P)/poly(hydroxybutyrate–co-hydroxyvalerate) (PHBV) and carbonated hydroxyapatite (CHAp)/poly (l-lactic acid) (PLLA) using SLS [148]. As mentioned earlier, the SLS induces controllable porosity within the melt pool surface [149]. Results of in vitro studies revealed that incorporation of nanocomposites improved SaOS-2 cell proliferation and alkaline phosphate activity. The authors proved that the nanocomposite scaffolds provided a biomimetic environment for osteoblastic cell attachment and have tremendous potential in bone tissue engineering applications. 

Qiyi Chen et al. prepared GO-based filaments for the FDM process [150]. GO was blended with thermoplastic polyurethane (TPU) and poly (lactic acid) (PLA) using solvent-based mixing process. The authors successfully used FDM to produce the nanocomposites, demonstrating their biocompatibility. The results showed that the mechanical properties could be improved proportionately with the increase of GO contents. In addition to the enhancement of mechanical properties, thermal stability was also improved with the presence of GO fillers. The in vitro results based on NIH_3_T_3_ cells showed that the 3D printed nanocomposites exhibited good biocompatibility and biological activities. 

When considering prosthetic devices due to birth defects, amniotic band syndrome is particularly common, occurring in one out of 1000 births approximately. The amniotic band syndrome affects limb malformation, commonly arms or hands [151,152]. Therefore, body-powered prosthetic bands are used due to their low cost, simplicity, and ease of maintenance compared to casted bionic prosthetics [153]. However, 3D printing offers personalized tissue prosthesis compared to conventional fabrication methods [154]. Thus, approaches for personalizing generic digital models of prosthetic components could enable the fabrication of low-cost personalized prostheses for children with amniotic band syndrome. Yuxin Tong et al. demonstrated that the combination of 3D scanning with 3D printing could enable the personalization of low-cost prosthetic hands with anatomically conformal electronic interfaces for children with amniotic band syndrome [155]. The authors observed that personalization of the prosthetic interface increased the tissue-prosthesis contact area by 408% relative to the non-personalized devices. Conformal 3D printing of carbon nanotube-based polymer inks across the personalized anatomical human-machine interface (AHMI) facilitated the integration of electronic components, specifically, conformal sensor arrays for measuring the pressure distribution across the AHMI (i.e., the tissue-prosthesis interface). Results revealed that the non-uniform pressure distribution across the AHMI was redistributed upon activation of the prosthetic hand’s grasping action. Subsequently, it seems critical to roughen the surface of the implant at the nanoscale level to increase the cellular response from the tissue. 

**Figure 5:**
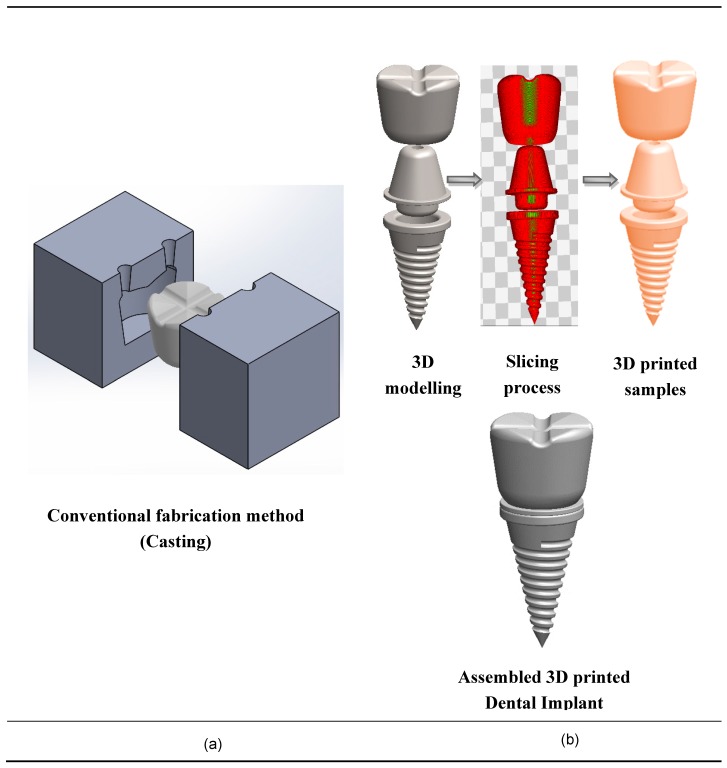
Fabrication techniques of dental implants. (**a**) Conventional fabrication techniques for dental implants; (**b**) 3D printing fabrication techniques for nanocomposite dental implants.

Finally, the use of AM technologies for dental applications has a huge potential due to complex geometries, low volumes, and high-degree of customization. Figure 5 shows the conventional fabrication method and additive manufacturing method, which validates that AM dental implants are more suitable for customization. Chang et al. [156] identified a novel device for scanning the denture image and subsequent reconstruction of 3D digital information of teeth models by abrasive computer tomography (ACT). 

## 6. Current Limitations in Additive Manufacturing of Implantable Devices 

The wide range of AM technologies currently available, from FDM to SLM or stereolithography, offers great versatility in the design and fabrication of complex biomedical implants and devices. Depending on the final application, specific techniques can be favored. For example, FDM is adapted for large-scale polymer-based objects (from 1 mm to ~50 cm), while stereolithography of UV-curable polymers can have resolutions below 1 mm [157]. The use of electrochemistry demonstrated printing resolutions as low as 250 nm [158]. In particular, SLM, a subset of AM, has rapidly evolved for certain applications such as tooling (conformal cooling) metals [159], aerospace structures [160], and the production of compact and complex functional metal parts [161,162]. However, AM technologies are limited by the small group of polymers and metal powders available. Moreover, in practical conditions, the product quality is such that the failure rates are quite high due to an improper understanding of the characteristics of the end-use product. 

As a consequence, AM technology still requires optimization to effectively 3D print bio-compatible functional components such as implantable devices. In addition, the materials involved for 3D printing of implantable devices are predominantly costly materials compared to those used in traditional manufacturing techniques. Thus, the technology sets limitations for the use of AM in sectors where high material integrity and sophistication are required. These issues have to be solved with advances in materials science and engineering i.e., by expanding the selection of materials and therefore lowering the cost. 

There are diverse parameters involved in AM metal processing which determine the attributes of the end products, such as material quality, layer thickness, laser or beam power, and gas flow. Optimizing different printing parameters will improve the 3D printing process [163,164]. This, however, makes the 3D printing of metal parts challenging, leading to time-consuming and costly processes. Simulation can be used to model the behavior of a part under a range of operating conditions and is now increasingly used to provide an understanding of the manufacturing process [163]. Despite these few limitations, 3D printing is expected to revolutionize medicinal fields, similar to the way the printing press transformed publishing [165].

## 7. Medical Industry Needs and Future Directions 

The medical/healthcare sector is one of the world’s fastest-growing industry [166], which consumes about 10% of the gross domestic product (GDP) of many developed nations [167]. Thus, the medical industry can form an enormous part of a country’s economy, leading to a potential funding market [168]. In addition, compared to other fields, the AM process is more compatible for the medical industry because complex bio-compatible components can be synergistically fabricated with minimal constraints and a high level of customization can be achieved. However, numerous factors such as repeatability, reliability, and seamless workflow must be considered to harvest all the benefits of AM techniques. The most complex and customized components, i.e., medical implants, can be processed in a faster and cheaper way through a sustainable AM route. It has been forecasted that the use of AM is estimated to grow at an annual rate of nearly 16% by 2020 [169]. Custom-made instruments and patient-specific implants may produce better outcomes in patients with abnormal anatomy, complex fractures, or neoplasms for whom traditional techniques are not compatible. In addition, if scaffolds with cellular products can be fashioned using bio printing techniques, then exact surface morphometry may be produced for a bio-implant and resurfacing of the joint. Irrespective of any type of medical application, it must be noted that the metal AM processes must be predictable and repeatable to supersede existing technology. Beyond all those technical factors discussed, it must be noted that the social policy [170] may assist in accomplishing further improvements and accelerating the commercialization of these technologies.

## 8. Conclusions

The use of nanomaterials in medical implants was introduced a few decades ago. However, their compatibility with emerging AM technologies remains challenging, in particular for niche medical applications. The present work provides an opportunity for researchers from various fields, from bioengineering to mechanical engineering, to have an insight into the selection of the 3D printing process appropriate materials depending on the type of application. A comprehensive assessment of the physics of material selection, process optimization, and design/geometry requirements will enable the rapid commercialization of AM technology for medical implant applications.

## Figures and Tables

**Table 1:** Micro and nanocomposites performed by AM medical implants.

Year	Implant	Materials	Micro/Nano	AM Method	Outcome Summary
**Microsized Materials**					
2000 [124]	Proposed for bone and dental	Titanium powder 200 μm and 60 μm	Micro	SLM	Fabricated dental crowns and bones with high strength and density
2003 [125]	Bone	PMMA	Micro	Proposed	Proposed the cost reduction Cranioplasty implants fabricated from AM using CT scanning image
2007 [126,127]	Bone	HA powder 2.78 μm	Micro	3DP Ink jet	Extensive bone ingrowth formation in 3D printed HA scaffolds
	Bone	Titanium alloy (Ti-6Al-4V)	Micro	SLM	Scaffolds are biocompatible, and pore width influences pore overgrowth, resistance to compressive force, and porosity.
2010 [128]	Tibial Knee stems, hip stems and intermedullary rods	Titanium alloys (Ti-6Al-4V) 100 μm	Micro	EBM	The array of cellular, reticular mesh manufactured in monolithic form has potential for unique bone compatibility
2012 [129]	Facial bone (orbital area)	Titanium (Ti64 Al4V-ELI) 30 μm	Micro	DMLS	The method enables exact fitting of implants, designed with low mass and therefore sensitive to hot and cold temperature
2013 [89]	Skull bone	polymer	Micro	SLS & Poly Jet	Fabricated skulls using Poly Jet and SLS, the accuracy of Poly Jet was higher than SLS or 3DP using novel measuring technique
2014 [130]	Bone (Cranial head)	Titanium (Ti64 ELI)	Micro	DMLS	Protocol developed and created an anatomic bio model of the bone defect for surgical planning and, finally, the design and manufacture of the patient-specific implant.
Nanosized Materials					
2008 [131]	Proposed for bone and dental	Titania nanotube	Nano	Proposed	Silver-treated Titania nanotube surface provides antibacterial properties to prevent implants against postoperative infections
2009 [132]	Endoscopic transplantation (oral muscular cells)	Poly(N-isopropylacrylamide) (PIPPAm)	Nano	EBM	Nanoscale thermo responsive surface to untimely reconstruct multifunctional three-dimensional tissues in vitro to regenerate a defective tissue
2015 [133]	Proposed for bone and dental	HA 100nm	Nano	Proposed	Synthesized HAp exhibits excellent biocompatibility,
2016 [134]	Bone grafting (Hip/Knee)	AgNPs- coated Ti6Al4V	Nano coating	EBM	Higher surface energy is observed for AgNPs-coated Ti6Al4V surfaces (70.17 mN/m) compared with uncoated ones (49.07 mN/m).
2017 [135]	Bone	AgNPs- coated Titanium (Ti-6Al-4V)	Nano coating	SLM	Antimicrobial assays consistently showed strong antimicrobial activity of the developed implants against MRSA including released activity, surface antimicrobial activity and prevention of biofilm formation.
2018 [136]	orthopedic	Silver nanoparticles (AgNPs)	Nano	Proposed	AgNP release, exploration of suitable size, shape, as well as the novel method of surface modification, such as 3DP technology
